# Overweight or Obesity and Outcomes in Children With Acute Lymphoblastic Leukemia

**DOI:** 10.1001/jamanetworkopen.2025.9952

**Published:** 2025-05-14

**Authors:** Elena J. Ladas, Haiyang Sheng, Uma H. Athale, Barbara L. Asselin, Luis A. Clavell, Peter D. Cole, Yael Flamand, Jean-Marie Leclerc, Caroline Laverdiere, Bruno Michon, Stephen E. Sallan, Lewis B. Silverman, Jennifer J. G. Welch, Song Yao, Kara M. Kelly

**Affiliations:** 1 Division of Pediatric Hematology, Oncology, and Stem Cell Transplantation, Columbia University Irving Medical Center, New York, New York; 2 Department of Cancer Prevention, Roswell Park Comprehensive Cancer Center, Buffalo, New York; 3 Division of Hematology-Oncology, McMaster Children’s Hospital, Hamilton Health Sciences, Hamilton, Ontario, Canada; 4 Department of Pediatrics, University of Rochester School of Medicine, Golisano Children’s Hospital at URMC, Rochester, New York; 5 Department of Pediatrics, San Jorge Children’s Hospital, San Juan, Puerto Rico; 6 Rutgers Cancer Institute of New Jersey, New Brunswick; 7 Division of Data Science, Dana-Farber Cancer Institute, Boston, Massachusetts; 8 Hematology-Oncology Division, Charles Bruneau Cancer Center, Sainte-Justine University Hospital, University of Montreal, Montreal, Quebec, Canada; 9 Centre Hospitalier Universitaire de Quebec, Sainte-Foy, Quebec, Canada; 10 Department of Pediatrics, Dana-Farber Cancer Institute, Boston, Massachusetts; 11 Division of Pediatric Hematology/Oncology, Hasbro Children’s Hospital, Brown University, Providence, Rhode Island; 12 Department of Pediatrics, Roswell Park Comprehensive Cancer Center and University at Buffalo Jacobs School of Medicine and Biomedical Sciences, Buffalo, New York

## Abstract

**Importance:**

There are conflicting data on the association of overweight or obesity with clinical outcomes in childhood acute lymphoblastic leukemia (ALL). The duration of exposure to overweight or obesity may be a better indicator of the risk of poorer outcomes.

**Objective:**

To determine the association of the duration of overweight or obesity with treatment-related toxic effects, minimal residual disease, relapse, and survival in childhood ALL.

**Design, Setting, and Participants:**

In this prospective cohort study, fluctuations in *z* scores of body mass index (BMI) for age from diagnosis to the end of treatment (EOT) were examined in 794 children registered on a Dana Farber Cancer Institute ALL Consortium protocol from May 31, 2005, to December 15, 2011. Height and weight were abstracted from the medical record for classification of BMI *z* scores at diagnosis through EOT and into survivorship. Data were analyzed from July 1 to 31, 2024.

**Main Outcomes and Measures:**

The duration of overweight or obesity was defined as having overweight or obesity at 2 or more time points and compared with having overweight or obesity at no more than 1 time point. Kaplan-Meier survival curves were generated to examine association of overweight or obesity with overall survival (OS), event-free survival (EFS), and cumulative incidence of relapse.

**Results:**

Among the 794 patients included in the analysis, the mean age at diagnosis was 6.7 (range, 1.0-17.9) years, with 441 (55.5%) being male, 136 (17.1%) Hispanic, and 553 (69.6%) non-Hispanic. The prevalence of overweight or obesity increased from 234 of 793 (29.5%) at diagnosis to 346 of 715 (48.4%) by EOT. Having overweight or obesity at baseline or developing overweight or obesity during induction was not associated with treatment-related toxic effects or higher minimal residual disease. Children with overweight or obesity at 2 or more time points experienced inferior OS (3-year OS, 93.8% vs 98.0%; *P* = .01), increased relapse (3-year relapse rate, 10.5% vs 5.8%; *P* = .02), and lower EFS (3-year EFS, 89.0% vs 93.7%; *P* = .02), compared with children with overweight or obesity at no more than 1 time point. Multivariable Cox proportional hazards regression models revealed an association between increased risk of death (hazard ratio [HR], 3.49; 95% CI, 1.28-9.51; *P* = .01) and relapse (HR, 1.92; 95% CI, 1.07-3.46; *P* = .03) among children with overweight or obesity at 2 or more time points.

**Conclusions and Relevance:**

In this prospective cohort study of children with ALL, longer duration of overweight or obesity was associated with lower OS and EFS and higher rates of relapse, underscoring the need for interventions targeting overweight or obesity during treatment of children with ALL.

## Key Points

**Question:**

Is the duration of overweight or obesity associated with survival in children with acute lymphoblastic leukemia (ALL)?

**Findings:**

In this cohort study of 794 children with ALL, longer exposure to overweight or obesity was associated with inferior overall survival and increased rate of relapse.

**Meaning:**

Evidence from this observational study suggests the need for interventions aimed at preventing the development of overweight or obesity during childhood ALL.

## Teaser

This cohort study examines the duration of overweight or obesity and its association with treatment-related toxic effects, the end of induction, minimal residual disease, relapse, and survival among children undergoing treatment for acute lymphoblastic leukemia.

## Introduction

Childhood overweight and obesity are persistent chronic health conditions affecting 16.1% of children aged 2 to 9 years and 19.3% of those aged 10 to 19 years.^1^ In children with acute lymphoblastic leukemia (ALL), the presence of overweight or obesity at diagnosis has been associated with an increased risk of several treatment-related toxic effects (TRTEs), including hepatic toxicity,^2,3,4^ hyperglycemia,^5^ and pancreatitis,^2,3^ as well as a higher risk of relapse^6^ and reduced survival.^7^ A large meta-analysis^7^ found that the presence of overweight or obesity at diagnosis was associated with an increased risk of mortality (relative risk [RR], 1.31; 95% CI, 1.09-1.58) and reduced event-free survival (EFS) (RR, 1.35; 95% CI, 1.20-1.51) among children with ALL.

In contrast, several studies^8,9,10^ have not observed an association of overweight or obesity at diagnosis with unfavorable outcomes. Rather, the duration of exposure to overweight or obesity has been hypothesized to confer an increased risk of unfavorable outcomes potentially due to the effect of adiposity on the leukemia microenvironment as well as the metabolome.^11^ While several studies have reported on fluctuations in body mass index (BMI) throughout treatment for childhood ALL,^12,13,14^ few have evaluated the association of fluctuations in BMI with clinical outcomes in ALL. Two studies performed in children with high-risk ALL and registered on a Children’s Oncology Group treatment study^3,15^ found that the duration of overweight or obesity was associated with an increased risk of relapse and death. Another study performed in Hispanic children with ALL undergoing treatment in Guatemala^16^ found that redressing poor nutritional status was associated with a reduced risk of death associated with poor nutritional status in the first 6 months of therapy. However, this has yet to be thoroughly examined in other risk groups or treatment regimens. We examined the duration of overweight or obesity and its association with TRTEs, the end of induction (EOI), minimal residual disease (MRD), relapse, and survival among children undergoing treatment for ALL and registered on the Dana Farber Cancer Institute ALL 05-001 Consortium (DFCI 05-001) clinical trial.^17^

## Methods

### Participants

Eligible participants were children and adolescents aged 1 to 18 years with newly diagnosed ALL who were enrolled in the DFCI 05-001 study from May 31, 2005, to December 15, 2011 (eTable 1 in Supplement 1). Consent to the treatment study was obtained as per the institutional review board of all participating centers and served as consent to this companion study. The details of this trial have been described elsewhere.^18,19,20^ This study followed the Strengthening the Reporting of Observational Studies in Epidemiology (STROBE) guidelines for cohort studies, and the STROBE flowchart for cohort studies is provided in eFigure 1 in Supplement 1.

### Study Design and Procedures

Details on study respondents and procedures have been previously published.^21^ Briefly, the Diet and Acute Lymphoblastic Leukemia Treatment cohort study (DALLT) is a prospective cohort study that collected demographic (investigator-reported ethnicity and race abstracted from medical records) and anthropometric (height and weight obtained by clinical personnel and reported in the electronic health record as per institutional standards) characteristics and caregiver-reported dietary intake survey data during a 2-year treatment period for ALL (diagnosis, EOI phase [approximately 32 days from diagnosis], and continuation phase [approximately 15 months after diagnosis]). A subset of patients (240 [30.2%]) were not able to provide anthropometric data at EOI due to limited personnel for medical record abstraction at several participating sites. Patients were identified as Asian, Black or African American, White, and other or unknown (including multiracial or not reported) race and as Hispanic, non-Hispanic, or not reported ethnicity. These race and ethnicity data were important to this study because of potential differences in obesity and survival outcomes.

Classification of BMI *z* score was performed according to the Centers for Disease Control and Prevention growth charts (underweight, *z* score less than −1.65; overweight, *z* score 1.04 or greater; obesity, *z* score 1.65 or greater).^22^ Key TRTEs were assessed in the treatment trial using National Cancer Institute Common Terminology Criteria for Adverse Events (version 3.0) and included the rates of infection (grade 3 or higher, including episodes of bacteremia or fungemia or radiographic evidence of invasive fungal infection), pancreatitis (grade 2 or higher), and thrombosis (grade 2 or higher) during and after induction through the end of treatment (EOT). Initial risk group was assigned at the time of study entry based on age, presenting leukocyte count, immunophenotype (B-cell vs T-cell), and central nervous system status based on established diagnostic criteria.^18,19,20^ MRD was assessed in those who achieved morphologic complete remission at EOI by real-time quantitative polymerase chain reaction analysis of patient-specific antigen receptor gene rearrangements as previously described.^23^ The final risk group for patients with B-cell ALL was assigned based on initial risk group, cytogenetics, and MRD results. Patients with B-cell ALL and indeterminate MRD results and all patients with T-cell ALL were assigned a final risk group based on initial risk group and cytogenetic findings. Clinical outcomes assessed were overall survival (OS) (survival without death from diagnosis to 5 years), cumulative incidence of relapse (fraction with relapse from diagnosis to 5 years), and EFS (survival without death or relapse from diagnosis to 5 years).

### Statistical Analysis

Data were analyzed from July 1 to 31, 2024. Descriptive statistics including mean and range for numeric variables and count and percentage for categorical variables were used to describe the patient population. Line plots were constructed to depict the trajectory of BMI *z* scores across the diagnosis, EOI, continuation, and EOT phases, overall and stratified by demographic variables, risk group, and baseline BMI *z* score classification. Paired *t* tests compared BMI *z* scores at diagnosis with those at later time points in therapy. To capture the time a patient spent in BMI *z* score at-risk categories (ie, overweight and obesity combined, with underweight and normal weight combined serving as the reference group) and the changes of moving in and out of these categories during treatment, patients were classified based on the number of time points with overweight or obesity at the diagnosis, continuation, and EOT phases. Due to a limited number of institutions reporting height and weight at EOI, BMI *z* scores at this time point were excluded from survival analysis. Kaplan-Meier survival curves from EOT to post treatment (5 years from study entry) for OS, relapse-free survival, and EFS were generated for patients with no more than 1 time point with overweight or obesity vs those with 2 or more time points with overweight or obesity, and *P* values were derived from a log-rank test. OS was defined as the time from diagnosis to the date of death, censored at the date of last follow-up. For cumulative incidence of relapse, only relapse is counted as an event.

In a different approach to classify the changes in at-risk BMI *z*-score categories during treatment, patients were grouped as having (1) underweight or normal weight at all 3 time points (reference group), (2) underweight or normal weight at diagnosis and then developing overweight or obesity at 2 or more time points, and (3) overweight or obesity at all 3 time points. Multivariable Cox proportional hazard models were used to derive hazard ratios (HRs) and 95% CIs for these groups of BMI *z* score changes, with adjustment for the final risk group, sex, and ethnicity determined a priori. For analysis of TRTEs (infections, pancreatitis, and thrombosis from EOI to EOT), the association between the occurrence of toxic effects at any time and the baseline BMI *z* score classification was tested using a χ^2^ test. In a subset of participants with data available, logistic regression was used to examine the association between the changes in BMI *z* scores with MRD status. All analyses were performed in R, version 4.2.1 (R Programing for Statistical Computing), and all tests were 2 sided, with an α level of .05 as cutoff for statistical significance.

## Results

### Patient Characteristics

A total of 794 eligible patients were enrolled in the DFCI 05-001 study, including 441 (55.5%) male and 353 (44.5%) female. Mean age was 6.7 (range, 1.0-17.9) years, and most were younger than 10 years (593 [75.7%]). Seventeen patients (2.1%) were Asian; 20 (2.5%), Black or African American; 412 (51.9%), White; and 345 (43.5%), other or unknown. One hundred thirty-six patients (17.1%) were Hispanic; 553 (69.6%), non-Hispanic; and 105 (13.2%), not reported. At diagnosis, 51 of 793 patients with data available (6.4%) were classified with underweight; 508 (64.1%), with normal weight; 124 (15.6%), with overweight; and 110 (13.9%), with obesity. These classifications reflect the nutritional status of healthy children in the US (Table 1).^1^ Most participants (697 [87.8%]) were diagnosed with B-cell ALL and 407 of 751 (54.2%) were classified as having standard risk disease for their final risk group.

**Table 1.** Demographic and Clinical Characteristics at Diagnosis

Variable	Patients, No. (%) (n = 794)
Age, mean (range), y	6.7 (1.0-17.9)
Age group	
<10 y	593 (74.7)
≥10 y	201 (25.3)
Sex	
Female	353 (44.5)
Male	441 (55.5)
Race	
Asian	17 (2.1)
Black or African American	20 (2.5)
White	412 (51.9)
Other or unknown^a^	345 (43.5)
Ethnicity	
Hispanic	136 (17.1)
Non-Hispanic	553 (69.6)
Not reported^b^	105 (13.2)
BMI *z* score classification at diagnosis^c^	
Underweight (less than −1.65)	51 (6.4)
Normal	508 (64.1)
Overweight (≥1.4)	124 (15.6)
Obesity (≥1.65)	110 (13.9)
Leukemia type	
B-cell	697 (87.8)
T-cell	97 (12.2)
Final risk group	
Standard risk	407 (54.2)
High risk	260 (34.6)
Very high risk	66 (8.8)
t(9;22) Translocation	18 (2.4)
Unknown	43
MRD status	
High	51 (7.8)
Low	497 (75.6)
Indeterminate	109 (16.6)
Unknown	137
Cranial radiotherapy	
Yes	193 (26.3)
No	540 (73.7)
Unknown	61

Abbreviations: BMI, body mass index; MRD, minimal residual disease.

^a^ Includes multiracial or not reported by clinical staff and/or patient.

^b^ Not reported by clinical staff and/or patient.

^c^ Height and weight were unknown for 1 patient.

### Trajectories of Changes in BMI *z* Scores During the Course of Treatment

**Figure 1.**
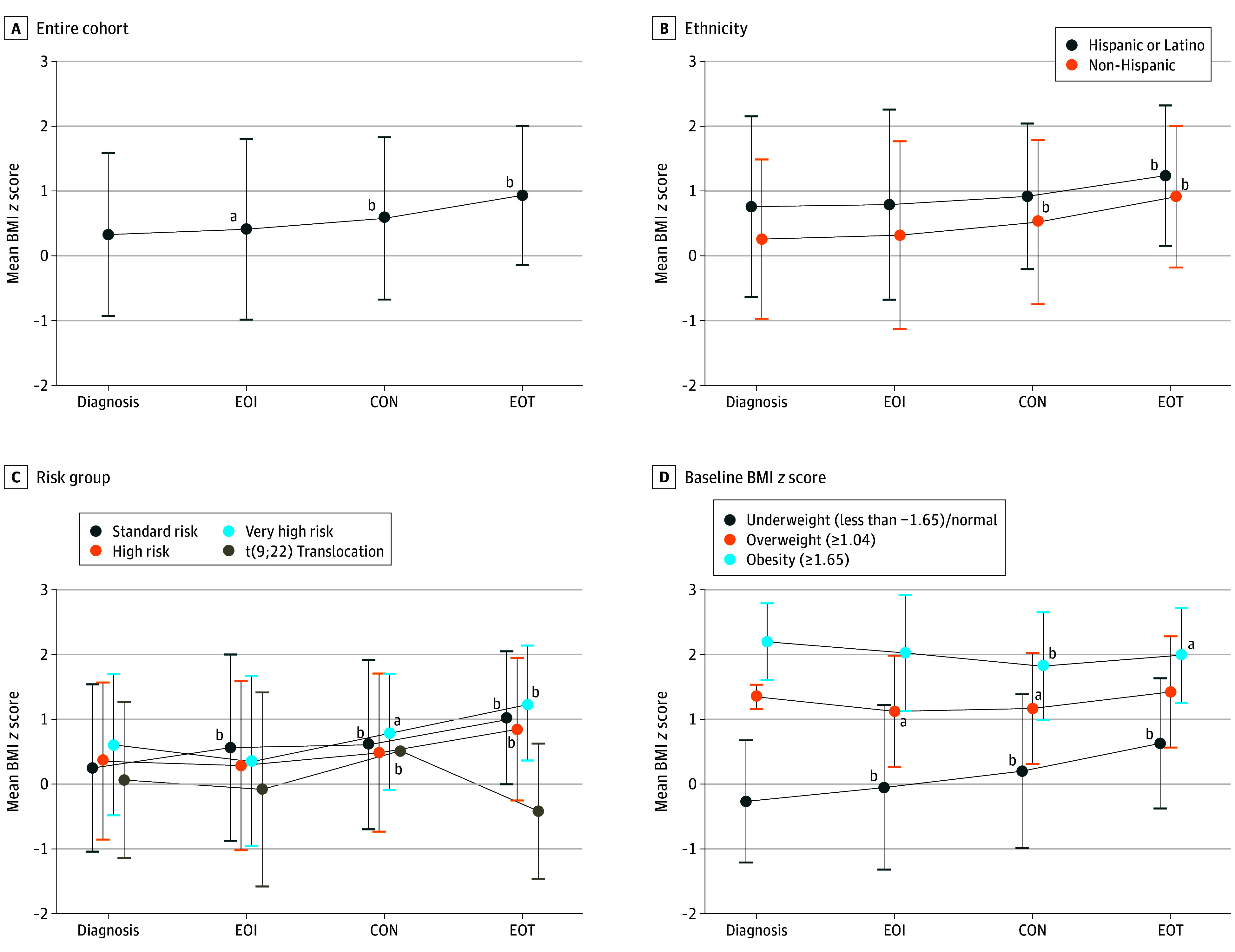
Mean Body Mass Index (BMI) *z* Score Trajectories From Diagnosis to End of Treatment (EOT) by Demographic and Clinical Characteristics Paired *t* test analyses were performed to compare the mean BMI *z* scores at diagnosis with those at end of induction (EOI), continuation (CON), and EOT. BMI is calculated as weight in kilograms divided by height in meters squared. ^a^*P* ≤ .05. ^b^*P* < .001.

eTable 2 in Supplement 1 presents the prevalence of nutritional status by time point. The prevalence of an underweight or normal weight BMI *z* score at diagnosis decreased from 559 of 793 (70.5%) at study entry to 369 of 715 (51.6%) by EOT (*P* < .001), which coincided with an increase in obesity from 110 of 793 (13.9%) at diagnosis to 186 of 715 (26.0%) at EOT (*P* = .02) and an increase in overweight from 124 of 793 (15.6%) and 160 of 715 (22.4%) (*P* = .28). The mean (SD) BMI *z* scores for the entire cohort increased from diagnosis (0.33 [1.26]) to EOI (0.41 [1.40]; *P* = .02), continuation (0.58 [1.25]; *P* < .001), and EOT (0.93 [1.07]; *P* < .001) (Figure 1A).

Further analyses of the trajectory of mean BMI *z* scores from diagnosis to EOT were conducted by stratifying by demographic and clinical variables (Figure 1B-D and eFigure 2 in Supplement 1). Only male patients experienced a significant increase in mean (SD) BMI *z* score early during treatment (diagnosis to EOI, mean [SD] to mean [SD]; [*P* = .01]), whereas both male and female patients experienced a significant increase in mean (SD) BMI *z* scores from diagnosis to continuation (male, 0.65 [1.30]; female, 0.49 [1.18]) and EOT (male, 0.92 [1.14]; female, 0.96 [0.99]) (*P* < .001 for all time points) (eFigure 2 in Supplement 1). The trajectory of increasing mean BMI *z* scores was similar between Hispanic and non-Hispanic children, although mean (SD) BMI *z* scores were higher among Hispanic patients at all 3 time points (EOI, 0.79 [0.47]; continuation, 0.92 [1.12]; EOT, 1.24 [1.08]), although increases only reached significance at diagnosis at EOT (*P* = .005) (Figure 1B). In analysis stratified by age at diagnosis (eFigure 2 in Supplement 1), significant increases in mean (SD) BMI *z* scores were observed for both patients younger than 10 years (EOI, 0.49 [1.41]; continuation, 0.65 [1.23]; EOT, 1.03 [1.00]; *P* < .001 for all time points) and at EOT only for patients 10 years or older (0.64 [1.23]; *P* = .001), with an initial decrease among older patients at EOI (0.14 [1.32]; *P* < .001). When stratified by ALL risk group (Figure 1C), children with standard risk experienced a persistent increase in mean (SD) BMI *z* scores throughout treatment (EOI, 0.56 [1.44]; continuation, 0.61 [1.31]; EOT, 1.02 [1.03]; *P* < .001 for all time points), whereas children with high risk experienced significantly higher mean (SD) BMI *z* scores only at EOT (0.84 [1.10]; *P* < .001) and children with very high risk experienced higher mean (SD) BMI *z* scores at continuation (0.81 [0.90]; *P* = .04) and EOT (1.25 [0.89]; *P* < .001) compared with BMI *z* scores at diagnosis. Last, we analyzed trajectories by BMI classification at diagnosis (Figure 1D). Children with an underweight or a normal weight BMI *z* score at diagnosis experienced the largest increase in mean (SD) BMI *z* scores (EOI, −0.05 [1.28]; continuation, 0.20 [1.19]; EOT, 0.63 [1.01]; *P* < .001 at all 3 time points). Children with overweight experienced a reduction in mean (SD) BMI *z* scores from diagnosis to EOI (1.12 [0.86]; *P* = .01) and continuation (1.17 [0.86]; *P* = .03); however, no significant difference was observed at EOT compared with mean (SD) BMI *z* scores at diagnosis (1.35 [0.19] vs 1.42 [0.86]; *P* = .28). In contrast, we observed a lower mean (SD) BMI *z* score at EOI (2.03 [0.90]; *P* = .19), continuation (1.82 [0.83]; *P* < .001), and EOT (1.99 [0.73]; *P* = .02) compared with BMI *z* scores at diagnosis for children with obesity (0.20 [0.59]). Due to the limited number of children with T-cell ALL, we performed sensitivity analysis within B-cell ALL, which showed a consistent pattern of significant increases in mean (SD) BMI *z* scores at EOI (0.43 [1.40]), continuation (0.61 [1.26]), and EOT (1.94 [1.06]) compared with the time of diagnosis (*P* < .001 for all 3 time points).

### Association of Obesity and Overweight With TRTE, MRD, Relapse, and Survival

#### Baseline BMI With Clinical Outcomes

Having overweight or obesity at diagnosis was not associated with risk of TRTEs, including bacteremia, pancreatitis, or thrombosis (eTable 3 in Supplement 1). Baseline BMI classification was not associated with EOI MRD (Table 2). Children with obesity at diagnosis appeared to have inferior OS compared with children with underweight or normal weight, although the difference did not reach statistical significance (3-year OS, 88.7% vs 95.7%; *P* = .09) (eFigure 3 in Supplement 1). No differences were found in cumulative incidence of relapse or EFS by baseline BMI classification (eFigure 3 in Supplement 1). In multivariable Cox proportional hazards regression models after adjustment for sex, ethnicity, and final risk group, children with overweight or obesity at diagnosis experienced a significantly higher risk of death than those with underweight or normal BMI (HR, 3.32; 95% CI, 1.18-9.35; *P* = .02).

**Table 2.** Association of MRD at End of Induction and BMI *z* Score at Diagnosis^a^

BMI *z* score classification at diagnosis	MRD results at end of induction, No. (%)			*P* value^a^
	Low (n = 496)	High (n = 51)	Indeterminate (n = 109)	
Underweight (less than −1.65) or normal weight	352 (71.0)	36 (70.6)	76 (69.7)	.95
Overweight (≥1.04)	76 (15.3)	8 (15.7)	15 (13.8)	
Obesity (≥1.65)	68 (13.7)	7 (13.7)	18 (16.5)	

Abbreviations: BMI, body mass index; MRD, minimal residual disease.

^a^ χ^2^ Test was performed comparing baseline BMI *z* score classification with end of induction MRD.

#### Association of Fluctuations in BMI With Clinical Outcomes

**Figure 2.**
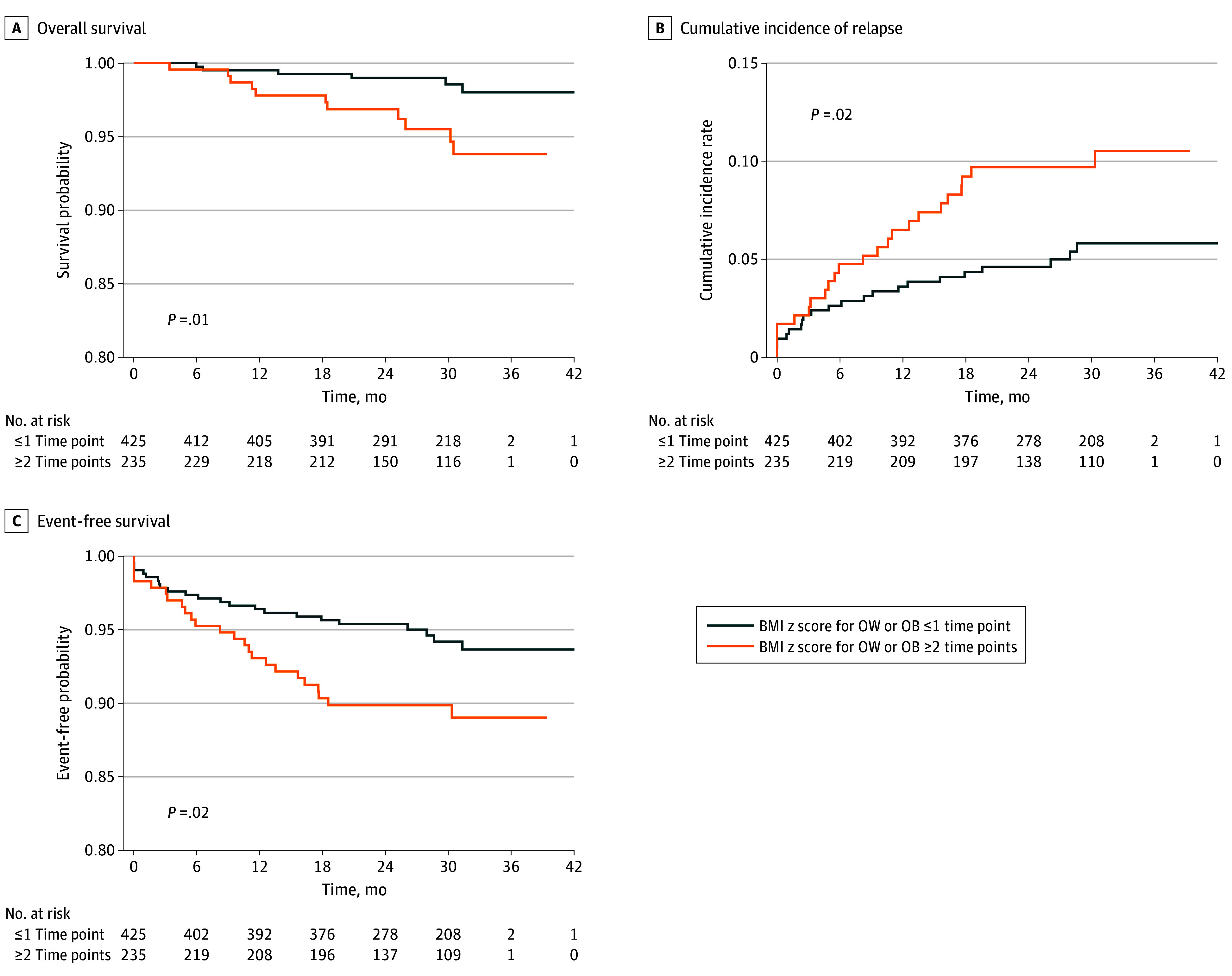
Overall Survival, Cumulative Incidence of Relapse, and Event-Free Survival From End of Treatment (EOT) to Post Treatment by Duration of Overweight (OW) or Obesity (OB) BMI indicates body mass index.

**Figure 3.**
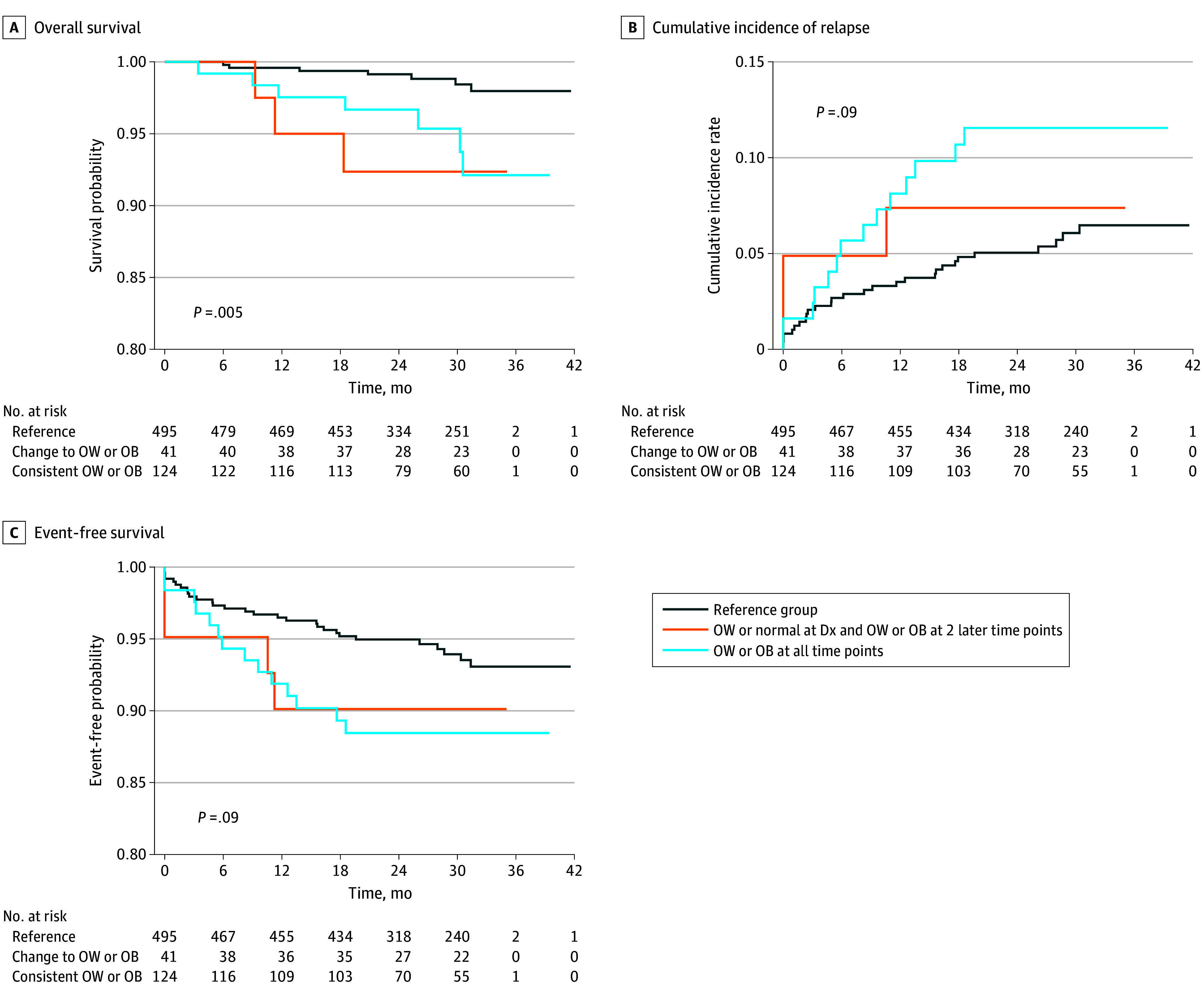
Overall Survival, Cumulative Incidence of Relapse, and Event-Free Survival From End of Treatment to Post Treatment by Number of Time Points With Overweight (OW) or Obesity (OB) Kaplan-Meier survival curves were generated comparing patients with an underweight or normal weight classification at all time points with patients with underweight or normal weight at diagnosis who developed OW or OB at 1 or more time point and patients with OW or OB at all 3 time points. Dx indicates diagnosis.

The mean (SD) follow-up time was 8.2 (3.6) years from diagnosis. Change in BMI classification from diagnosis to EOI was not associated with EOI high MRD. Compared with children with overweight or obesity at no more than 1 time point, those with overweight or obesity at 2 or more time points during therapy experienced significantly inferior OS (3-year OS, 93.8% vs 98.0%; *P* = .01), higher cumulative incidence of relapse (3-year relapse rate, 10.5% vs 5.8%%; *P* = .02), and lower EFS (3-year EFS, 89.0% vs 93.7%; *P* = .02) (Figure 2). Compared with children with underweight or normal weight at all 3 time points, those who developed overweight or obesity after diagnosis or children with overweight or obesity at all 3 time points had similarly higher incidence of death (3-year OS, 98.0%, 92.4%, and 92.1%, respectively; *P* = .01), cumulative incidence of relapse (3-year relapse rate, 6.5%, 7.4%, and 11.6%, respectively; *P* = .09), and EFS (3-year EFS, 93.1%, 90.1%, and 88.5%, respectively; *P* = .09) (Figure 3), although the latter 2 comparisons did not reach statistical significance.

Multivariable Cox proportional hazards regression models with adjustment for sex, ethnicity, and final risk group revealed an association of an increased risk of death among children with overweight or obesity at 2 or more time points (HR, 3.49; 95% CI, 1.28-9.51; *P* = .01). Similar results were observed for cumulative incidence of relapse (HR, 1.92; 95% CI, 1.07-3.46; *P* = .03) and EFS (HR, 1.92; 95% CI, 1.08-3.41; *P* = .03) (eTable 4 in Supplement 1). These increased risks remained when further adjusting for baseline BMI classification in the model, although the increase was only statistically significant for EFS (HR, 2.42; 95% CI, 1.08-5.39; *P* = .03).

## Discussion

Prior studies have found that BMI for age is associated with relapse, survival, and TRTEs in children with ALL, although inconsistently.^9,24,25,26^ Most studies have relied on BMI for age at diagnosis^6,8,10,27^; few have evaluated fluctuations in BMI. In this analysis of a large prospective cohort receiving uniform treatment through the DFCI 05-001 phase 3 study, we found that the duration of overweight and obesity was associated with relapse and survival.

The duration of exposure to an at-risk BMI has been previously reported in 2 studies^3,15^ among children with high-risk ALL registered on Children’s Oncology Group protocols and in 1 study among children with standard-risk and high-risk ALL undergoing treatment in a low- and middle-income setting.^16^ Orgel et al^3^ (N = 2008) evaluated the association of BMI with event-free survival during a median follow-up of 8.5 years, whereas Wadhwa et al^15^ (N = 676) evaluated the association of BMI with the risk of relapse during median follow-up of 6 years. Antillon et al,^16^ the only study conducted in a low- and middle-income setting, evaluated the association of BMI with 6-month survival among 331 children with high- and low-risk ALL. Each of these studies reported an association of poor nutritional status with an increased risk of death or relapse. Our study serves as a validation of previous studies by examining the association of overweight and obesity with clinical outcomes in children registered on a DFCI 05-001 protocol and expands these findings to include children with standard-risk ALL. Despite higher cumulative exposure to corticosteroids in DFCI 05-001 ALL regimens, the prevalence of overweight or obesity appears to be similar to rates reported with other ALL treatment protocols, suggesting other factors may also contribute to the development of overweight or obesity during treatment for ALL. Collectively, these studies suggest the need for interventions addressing the prevention of overweight or obesity during treatment for childhood ALL among those who do not have overweight or obesity at diagnosis as well as the development of treatment strategies for children with overweight or obesity at diagnosis that persists throughout treatment. Ongoing efforts targeting the prevention of overweight and obesity in childhood ALL are under way.^28^

In contrast to a small single-institution study consisting mostly of Hispanic children with overweight and obesity,^26^ we did not observe an association between overweight or obesity and EOI MRD. Additional research in heterogeneous populations and varied treatment protocols that advance our understanding of the underlying mechanisms of overweight and obesity in childhood ALL may offer insight into these conflicting observations. We did not find an association with toxic effects, which is in contrast to a previous study reporting on BMI classification at diagnosis among adolescents and young adults (aged 15-50 years) treated with a comparable DFCI 05-001 ALL regimen.^4^ In contrast to weight gain occurring during treatment, persistent excess weight prior to and during the early phases of treatment may impact the development of the leukemic microenvironment, conferring protection from the treatment itself and altering drug pharmacokinetics.^11,29^

Our results are consistent with previous analyses^12,14^ in that BMI *z* score increases significantly during the duration of treatment (Table 2). Previous studies have reported the prevalence of overweight and obesity at EOT to range from 22%^30^ to 70%,^31^ with geographic region and demographic characteristics contributing to the variations in findings. A previous study^32^ suggested that the prevalence of overweight and obesity among survivors of ALL is aligned with national data, and concern is not warranted; our results suggest otherwise. More recent data^33^ suggest that survivors of childhood ALL who live in North America experience a higher prevalence of overweight and obesity relative to survivors of ALL in Europe, which may be due to a combination of lifestyle, varying treatment protocols, genetics, or other nonidentified variables.

Younger age,^34,35,36^ female sex,^34,37,38,39^ male sex,^12,40^ and Hispanic ethnicity^41^ have been identified as risk factors for the development of overweight and obesity in some but not all studies.^14,35^ Among Hispanic children in the present study, increases in BMI *z* scores occurred later in treatment, perhaps due to the already high prevalence of overweight and obesity (64.6%) among Hispanic children at diagnosis. Several hypotheses for weight gain during childhood ALL have been put forth. Type, dose, and duration of corticosteroids, a core component of treatment for ALL, have been proposed; however, this does not explain the group of children who avoid weight gain entirely. Previous studies including investigators from our group have shown that dietary quality^42^ and the microbiome^43^ may be contributing factors while others have suggested that genetic predisposition may explain up to 6.2% of obesity among survivors of childhood ALL.^44^ Our significant observations in death and relapse, rather than toxic effects, suggests that dose reductions or delays in treatment may not contribute to the poor survival in this group of patients. While previous studies have found older age,^45,46^ Hispanic ethnicity,^47^ and obesity^2^ to be associated with pancreatitis, it is notable that we did not observe this association in our cohort. The lower mean age of this cohort and the limited sample size to perform subset analysis may explain the lack of association.

Multiple biological, social, and behavioral factors associated with obesity may contribute to treatment failure in children with ALL.^11^ An increased prevalence of high-risk B-cell ALL with rearrangements in the cytokine receptor–like factor 2 (*CRLF2r*) ALL has been observed in children with obesity, suggesting that obesity may confer a higher risk of poor prognosis subtypes, although this would not explain our observations of inferior survival outcomes for those who develop overweight or obesity during treatment.^48^ Biological pathways that have linked obesity with poorer outcomes to ALL therapy include the presence of adipose tissue in the bone marrow; the effect of insulin, the insulinlike growth factor system, and sex hormones; the presence of proinflammatory cytokines, including interleukin 6 and tumor necrosis factor; and adipocytokines such as adiponectin, leptin, resistin, and visfatin; dyslipidemia and lipid signaling; and chronic low-grade inflammation and oxidative stress.^49^ These may lead to alterations in the leukemia microenvironment, altering the effects of chemotherapy. The impact of social factors such as structural racism likely contribute to increased mortality risk.^50^

### Strengths and Limitations

The strengths of our study include the prospective collection of nutritional data at multiple time points across varying intensities of chemotherapy administration. However, our study has some limitations. BMI *z* scores are not an ideal indicator of nutritional status, particularly adiposity. Previous studies in children with ALL^51,52^ have suggested that BMI *z* scores may underestimate adiposity in children with ALL, potentially misclassifying some of the children in our cohort. Our large cohort of several groups such as Hispanic patients and those sex and risk groups who may be especially susceptible to weight gain was a strength; however, the sample size of these groups limited the statistical power for subgroup analyses of the associations as well as associations of overweight and obesity with TRTE and MRD. Stratification of BMI *z* scores by several of these variables addresses some of these limitations, but not all. Our results must consider that we were not able to collect information on some variables that affect overweight and obesity in children, including stage of pubertal status, physical activity, and food security, which will be addressed in some ongoing studies. The impact of the duration of exposure to overweight or obesity or the timing of its development (early vs later in treatment) may be analyzed in larger cohorts. Several global initiatives are examining these factors in children with ALL exposed to varying environmental and lifestyle factors, and treatment protocols are under way.^53,54^ In addition, our results align with those of a previous study^36^ performed among children receiving treatment with prior DFCI 05-001 ALL regimens, even with the reduction in the percentage of children receiving cranial radiotherapy in contemporary treatment regimens.

## Conclusions

In this cohort study, the duration of overweight or obesity was adversely associated with relapse and survival in childhood ALL. Considering that overweight or obesity is a modifiable risk factor, our results underscore the need for interventions aimed at avoiding new-onset overweight or obesity as well as treatment-based interventions for children with overweight or obesity at diagnosis and throughout treatment.

## Notes

Supplement 1. **eTable 1.** DFCI 05-001 Treatment Protocol **eTable 2.** Prevalence of Weight Classification (BMI *z* Score) by Time Point **eTable 3.** Association of Treatment-Related Toxicity With BMI *z* Score Classification at Diagnosis **eTable 4.** Overall Survival, Relapse-Free Survival, and Event-Free Survival Among Children With OW or OB on 2 or More Time Points From Diagnosis to EOT **eFigure 1.** STROBE Flowchart **eFigure 2.** Mean BMI *z* Score Trajectories From Diagnosis to EOT by Demographic and Clinical (ALL and Nutrition Status) Characteristics **eFigure 3.** Overall Survival, Cumulative Incidence of Relapse, and Event-Free Survival From EOT to Post Treatment by BMI *z* Score Classification at Diagnosis

Supplement 2.
Data Sharing Statement
