# Supplementary material for: Overweight or Obesity and Outcomes in Children With Acute Lymphoblastic Leukemia
Source: JAMA Netw Open. 2025 May 14;8(5):e259952. doi: 10.1001/jamanetworkopen.2025.9952 (PMC12079296; doi:10.1001/jamanetworkopen.2025.9952)
Supplement: Supplement 1. — eTable 1. DFCI 05-001 Treatment Protocol eTable 2. Prevalence of Weight Classification (BMI z Score) by Time Point eTable 3. Association of Treatment-Related Toxicity With BMI z Score Classification at Diagnosis eTable 4. Overall Survival, Relapse-Free Survival, and Event-Free Survival Among Children With OW or OB on 2 or More Time Points From Diagnosis to EOT eFigure 1. STROBE Flowchart eFigure 2. Mean BMI z Score Trajectories From Diagnosis to EOT by Demographic and Clinical (ALL and Nutrition Status) Characteristics eFigure 3. Overall Survival, Cumulative Incidence of Relapse, and Event-Free Survival From EOT to Post Treatment by BMI z Score Classification at Diagnosis [file jamanetwopen-e259952-s001.pdf]

Supplementary Online Content

Ladas EJ, Sheng H, Athale UH, et al. Overweight or obesity and outcomes in children with acute lymphoblastic leukemia. *JAMA Netw Open.* 2025;8(X):e259952. doi:10.1001/jamanetworkopen.2025.9952

eTable 1. DFCI 05-001 Treatment Protocol
eTable 2. Prevalence of Weight Classification (BMI *z* Score) by Time Point
eTable 3. Association of Treatment-Related Toxicity With BMI *z* Score Classification at Diagnosis
eTable 4. Overall Survival, Relapse-Free Survival, and Event-Free Survival Among Children With OW or OB on 2 or More Time Points From Diagnosis to EOT
eFigure 1. STROBE Flow Chart
eFigure 2. Mean BMI *z* Score Trajectories From Diagnosis to EOT by Demographic and Clinical (ALL and Nutrition Status) Characteristics
eFigure 3. Overall Survival, Cummulative Incidence of Relapse, and Event-Free Survival From EOT to Post Treatment by BMI *z* Score Classification at Diagnosis

This supplementary material has been provided by the authors to give readers additional information about their work.

© 2025 Ladas EJ et al. *JAMA Network Open*.

eTable 1. DFCI 05-001 Treatment Protocol[1]

Phase	Induction	Consolidation I	CNS	Consolidation II	Continuation
	**All patients**	**All patients**	**SR**	**SR#**	**All patients**
**Treatment Details**	*Steroid Prophase (Days 1-3):* Methlyprednisone 32 mg/m^2 IV divided three times daily, days 1-3<br>*Remission Induction Therapy (Days 4-32):*<br>- Vincristine (VCR) 1·5 mg/m^2 IV once per week (maximum 2 mg), days 4, 11, 18, and 25<br>- Prednisone 40 mg/m^2 PO divided three times daily, days 4 to 32<br>- Doxorubicin (DOX) 30 mg/m^2 IV, days 4 and 5<br>- Methotrexate (MTX) 40 mg/m^2 IV push, day 6<br>- Pegasparaginase (IV-PEG) 2,500 IU/m^2 IV, day 7<br>*Intrathecals:* IT cytarabine dosed by age on day 0†; IT methotrexate/cytarabine/hydrocortisone (IT-MAH) dosed by age on day 18; IT-methotrexate (ITM) dosed by age on day 32	- VCR 2 mg/m^2 (maximum 2 mg) IV, day 1<br>- 6-mercaptopurine (6-MP) 50 mg/m^2 PO daily at bedtime, days 1-14<br>- ITM dosed by age, day 1‡<br>- High dose MTX 5 grams/m^2 IV over 24 hours on day 1, followed by Leucovorin rescue	- VCR 2 mg/m^2 (maximum 2 mg) IV, day 1<br>- 6-MP 50 mg/m^2 PO daily at bedtime, days 1-14<br>- DEX 18 mg/m^2 PO divided twice daily, days 1-5<br>- Start ASP according to randomization starting on day 1‡‡	Every 3-week cycles:<br>- VCR 2 mg/m^2 (maximum 2 mg) IV, day 1<br>- 6-MP 50 mg/m^2 PO daily at bedtime, days 1-14<br>- DEX 6 mg/m^2 PO divided twice daily, days 1-5<br>- MTX 30 mg/m^2 IV push or IM weekly, days 1, 8, and 15 (Week 1 MTX omitted if IT-MAH given)<br>- IT-MAH dosed by age every nine weeks for six doses and then every 18 weeks<br>- ASP continued according to randomization‡‡	Every 3-week cycles:<br>- VCR 2 mg/m^2 (maximum 2 mg) IV, day R<br>- 6-MP 50 mg/m^2 PO daily at bedtime, days 1-14<br>- DEX 6 mg/m^2 PO divided twice daily, days 1-5<br>- MTX 30 mg/m^2 IV push or IM weekly, days 1, 8, and 15 (Week 1 MTX omitted if IT-MAH given)<br>- IT-MAH dosed by age every 18 weeks
	**Ph + ALL ††**	**HR and VHR**	**HR and VHR**	**HR and VHR**	
**Treatment Details**	Imatinib 340 mg/m PO (maximum 600 mg) daily starting on day 18	Same as SR patients with the addition of:<br>DOX 30 mg/m^2 IV on day 1 with dexrazoxane 300 mg/m^2 IV	Same as SR, except:<br>- DOX 30 mg/m^2 IV on day 1 with dexrazoxane 300 mg/m^2 IV<br>- DEX 18 mg/m^2 PO divided twice daily, days 1-5<br>- HR patients: Start ASP according to randomization starting on day 1‡‡	Every 3-week cycles, same as SR, except:<br>- No weekly MTX<br>- DOX 30 mg/m^2 IV on day 1 with dexrazoxane 300 mg/m^2 IV<br>- Once cumulative DOX reaches 300 mg/m^2 IV, weekly MTX given as in the SR arm<br>- DEX 18 mg/m^2 PO divided twice daily, days 1-5	

© 2025 Ladas EJ et al. *JAMA Network Open*.

		**VHR**	**Cranial radiation**	**Cranial radiation**	
**Treatment Details**		In addition to above:<br>Consolidation IB<br>- Cyclophosphamide 1000 mg/m^2 IV over 1 hour, day 1<br>- Cytarabine 75 mg/m^2 IV push daily, days 2-5 and 9-12<br>- 6-MP 50 mg/m^2 PO daily at bedtime, days 1-14<br>- ITM dose by age, day 1<br>Consolidation IC<br>- Cytarabine 2 grams/m^2 IV every 12 hours for a total of four doses starting on day 1<br>- Etoposide 100 mg/m^2 IV daily, days 3-5<br>- Dexamethasone (DEX) 18 mg/m^2 PO divided twice daily, days 1-5 (10 doses total)<br>- Asparaginase (ASP) according to randomization, beginning on day 8‡‡	- None: All SR, HR B-ALL with presenting WBC < 100K/□1 and absence of CNS-3<br>- 12 Gy: CNS-1 or CNS-2 with any of the following: T-ALL, VHR group, or B-ALL with WBC ≥ 100K/□1<br>- 18 Gy: CNS-3 at diagnosis	- None: IT-MAH dosed by age every nine weeks for six doses, and then every 18 weeks<br>- Prior cranial radiation: IT-MAH every 18 weeks	
				Consolidation II ends after 30 weeks of asparaginase therapy and cumulative DOX dose of 300 mg/m^2.	Treatment completed after 104 weeks of continuous complete remission.

[1]Adapted from Place AE, et al. *Lancet Oncology* November 6, 2015.
Abbreviation: IV: intravenous; PO: by mouth
† Patients with CNS leukemia at diagnosis (CNS-2 and CNS-3) received IT cytarabine two times per week until CSF clear of blast cells on three consecutive examinations.
†† Patients with Ph+ ALL continued to receive imatinib in combination with HR chemotherapy until they proceeded to stem cell transplant. These patients did not participate in asparaginase randomization and received IM-EC during post-induction therapy.
‡ ITM not administered if Day 32 ITM given within previous 72 hours
‡‡ IM-EC 25,000 IU/m^2/ dose IM x every week (30 consecutive doses total) or IV-PEG 2,500 IU/m^2/ dose IV every two weeks (15 consecutive doses total).
# SR patients who received fewer than 10 weeks of post-induction asparaginase due to toxicity were treated with three cycles of “HR” Consolidation II therapy, including additional doxorubicin (to cumulative dose 150 mg/m^2) and higher dose dexamethasone. No changes to treatment were made for HR/VHR patients who tolerated fewer than 10 weeks of post-induction asparaginase.

© 2025 Ladas EJ et al. *JAMA Network Open*.

© 2025 Ladas EJ et al. *JAMA Network Open*.

eTable 2. Prevalence of Weight Classification (BMI *z* Score) by Time Point*

	**BMIz Status Study Entry (N/%)**	**BMIz Status EOI (N/%)**	**BMIz Status CON (N/%)**	**BMIz Status EOT (N/%)**
**Underweight/Normal**	559 (70.5%)	367 (66.2%)[a]	452 (63.5%)[a]	369 (51.6%)[a]
**Overweight**	124 (15.6%)	84 (15.2%)[b]	126 (17.7%)[c]	160 (22.4%)
**Obese**	110 (13.9%)	103 (18.6)	134 (18.8%)[a]	186 (26.0%)[d]
**Total**	793	554	712	715

*Paired t-test were carried out to compare the mean BMIz scores at study entry with each of the following 3 time points. [a]$P < 0.001$; [b]$P < 0.01$; [c]$P < 0.03$; [d]$P < 0.02$

eTable 3. Association of Treatment-Related Toxicity With BMI *z* Score Classification at Diagnosis*

**Outcome**	**BMI Classification at Diagnosis***			
	Underweight/Normal N=559	Overweight N=124	Obese N=110	*P* value
Treatment-Related Toxicities**	322 (58%)	73 (59%)	67 (61%)	0.80

*Chi-squre tests for contingency table. **TRT includes children who developed either infection, pancreatitis, or thrombosis by EOT.

eTable 4. Overall Survival, Relapse-Free Survival, and Event-Free Survival Among Children With OW or OB on 2 or More Time Points From Diagnosis to EOT

Outcome	Hazard Ratio, 95% CI*	*P* value	Hazard Ratio, 95% CI**	*P* value
Overall survival	3.49 (1.28, 9.51)	0.01	3.42 (0.79-14.86)	0.10
Relapse-free survival	1.92 (1.07, 3.46)	0.03	2.15 (0.93-4.96)	0.07
Event-free survival	1.92 (1.08, 3.41)	0.03	2.42 (1.08-5.39)	0.03

*Multivariable Cox model adjusted for final risk group, ethnicity, and sex; **Multivariable Cox model adjusted for final risk group, ethnicity, sex, and baseline BMI.

© 2025 Ladas EJ et al. *JAMA Network Open*.

eFigure 1. STROBE Flow Chart

STROBE, Strengthening the Reporting of Observational Studies in Epidemiology.

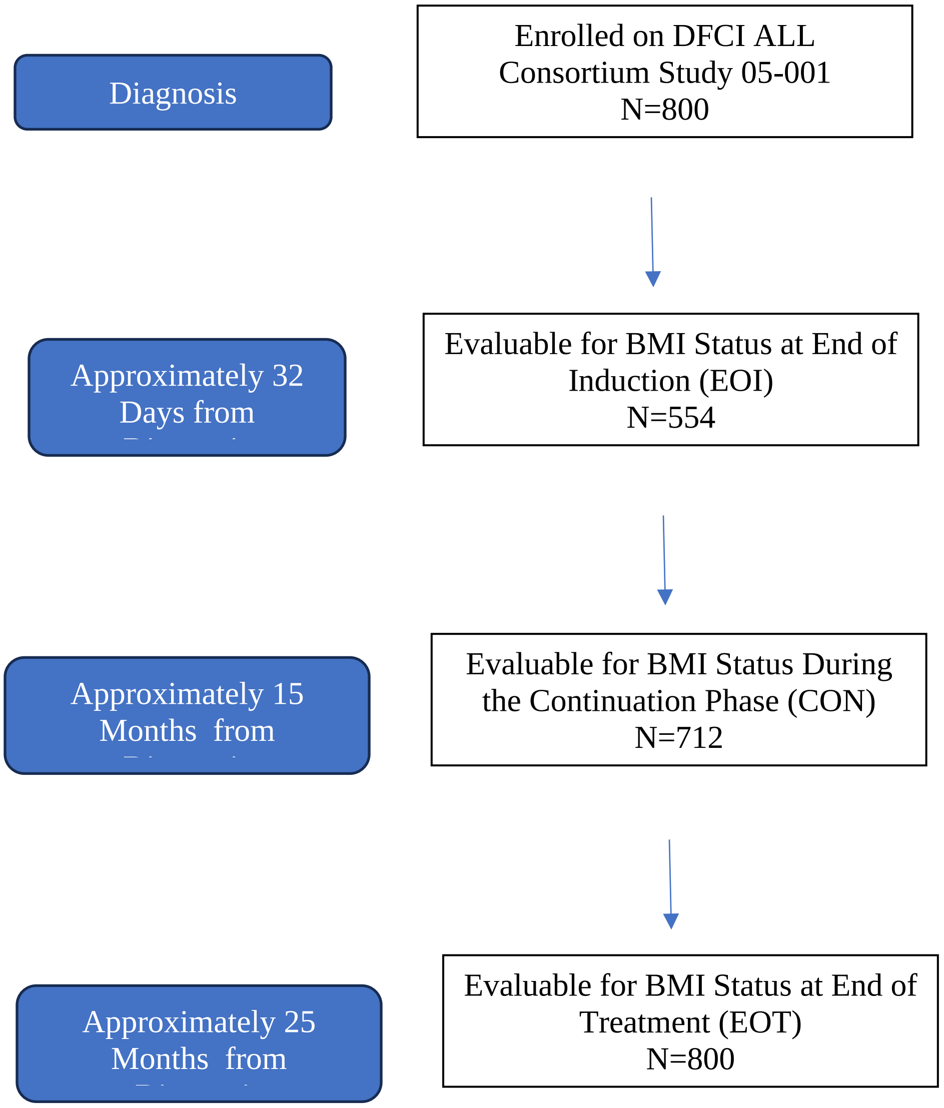

© 2025 Ladas EJ et al. *JAMA Network Open*.

eFigure 2. Mean BMI *z* Score Trajectories From Diagnosis to EOT by Demographic and Clinical (ALL and Nutrition Status) Characteristics

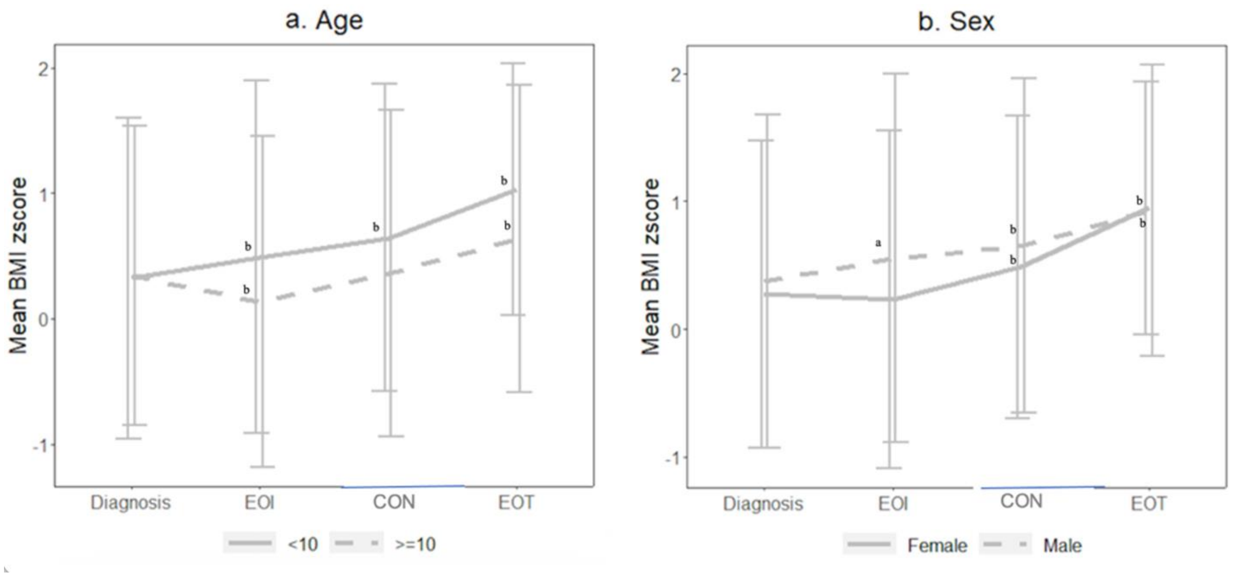

*Paired t-test were carried out to compare the mean BMIz scores of dx with each of the following 3 timepoints, respectively. [a]$P \leq 0.05$; [b]$P < 0.001$

© 2025 Ladas EJ et al. *JAMA Network Open*.

eFigure 3. Overall Survival, Cummulative Incidence of Relapse, and Event-Free Survival From EOT to Post Treatment by BMI *z* Score Classification at Diagnosis*

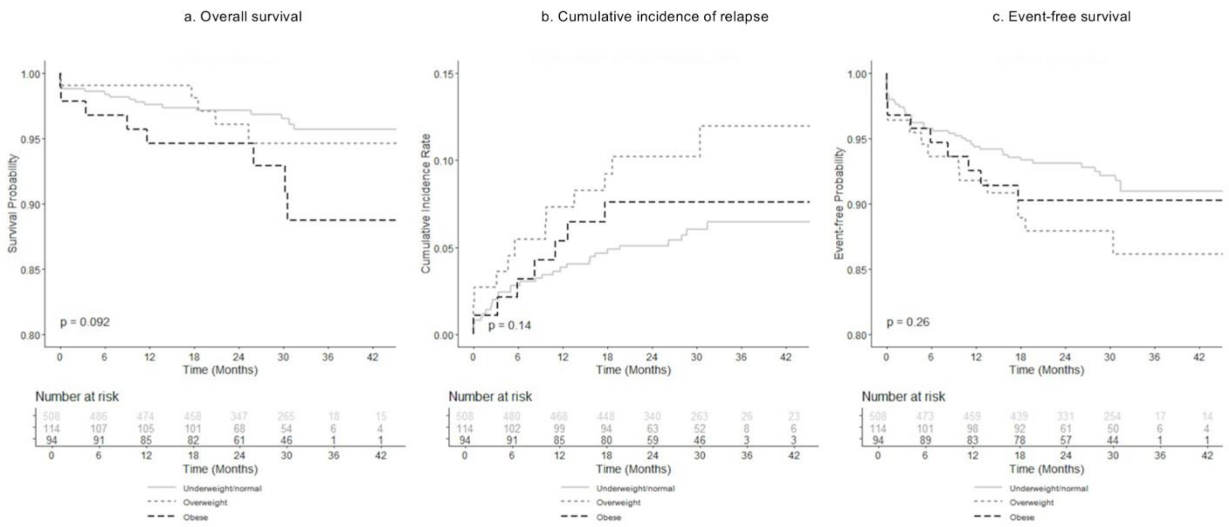

* Kaplan-Meier survival curves were generated comparing baseline BMI classification to overall survival, relapse, and event-free survival.

© 2025 Ladas EJ et al. *JAMA Network Open*.
