# Supplementary material for: Overweight or Obesity and Outcomes in Children With Acute Lymphoblastic Leukemia
Source: JAMA Netw Open. 2025 May 14;8(5):e259952. doi: 10.1001/jamanetworkopen.2025.9952 (PMC12079296; doi:10.1001/jamanetworkopen.2025.9952)
Supplement: Supplement 2. — Data Sharing Statement [file jamanetwopen-e259952-s002.pdf]

Data Sharing Statement

Ladas. Overweight or Obesity and Outcomes in Children With Acute Lymphoblastic Leukemia. *JAMA Netw Open*. Published May 14, 2025. doi:10.1001/jamanetworkopen.2025.9952

Data
Data available: Yes
Data types: Deidentified participant data
How to access data: Please email ejd14@cumc.columbia.edu
When available: With publication

Supporting Documents
Document types: None

Additional Information
Who can access the data: researchers with an approved proposal by the PI of the study
Types of analyses: specified purpose
Mechanisms of data availability: signed data access agreement
